# Hypoxia promotes redifferentiation and suppresses markers of hypertrophy and degeneration in both healthy and osteoarthritic chondrocytes

**DOI:** 10.1186/ar4272

**Published:** 2013-08-21

**Authors:** Brandon D Markway, Holly Cho, Brian Johnstone

**Affiliations:** 1 Department of Orthopaedics & Rehabilitation, Oregon Health & Science University, 3181 SW Sam Jackson Park Road, OP31, Portland, OR 97239 USA

## Abstract

**Introduction:**

Hypoxia is considered to be a positive influence on the healthy chondrocyte phenotype and cartilage matrix formation. However, hypoxia-inducible factors (HIFs) have been implicated in the pathogenesis of osteoarthritis (OA). Thus, we assessed whether healthy and OA chondrocytes have distinct responses to oxygen, particularly with regard to hypertrophy and degradation during redifferentiation.

**Methods:**

Monolayer-expanded healthy and OA chondrocytes were redifferentiated for 14 days in pellet cultures under standard (20% oxygen) or hypoxic (2% oxygen) conditions. Cartilage matrix gene expression, matrix quality and quantity, degradative enzyme expression and HIF expression were measured.

**Results:**

In hypoxia, both healthy and OA chondrocytes had higher human collagen type II, α1 gene (*COL2A1*), and aggrecan (*ACAN*) expression and sulfated glycosaminoglycan (sGAG) accumulation, concomitant with lower human collagen type X, α1 gene (*COL10A1*), and human collagen type I, α1 gene (*COL1A1*), expression and collagen I extracellular accumulation. OA chondrocytes had significantly lower sGAGs/DNA than healthy chondrocytes, but only in high oxygen conditions. Hypoxia also caused significantly greater sGAG retention and hyaluronic acid synthase 2 (*HAS2*) expression by OA chondrocytes. Both healthy and OA chondrocytes had significantly lower expression of matrix metalloproteinases (MMPs) *MMP1*, *MMP2*, *MMP3 *and *MMP13 *in hypoxia and less active MMP2 enzyme, consistent with lower *MMP14 *expression. However, aggrecanase (*ADAMTS4 *and *ADAMTS5*) expression was significantly lowered by hypoxia only in healthy cells, and *COL10A1 *and *MMP13 *remained significantly higher in OA chondrocytes than in healthy chondrocytes in hypoxic conditions. HIF-1α and HIF-2α had similar expression profiles in healthy and OA cells, increasing to maximal levels early in hypoxia and decreasing over time.

**Conclusions:**

Hypoxic culture of human chondrocytes has long been acknowledged to result in increased matrix accumulation, but still little is known of its effects on catabolism. We show herein that the increased expression of matrix proteins, combined with decreased expression of numerous degradative enzymes by hypoxia, minimizes but does not abolish differences between redifferentiated healthy and OA chondrocytes. Hypoxia-induced HIF expression is associated with hypertrophic marker and degradative enzyme downregulation and increased measures of redifferentiation in both healthy and OA chondrocytes. Therefore, though HIFs may be involved in the pathogenesis of OA, conditions that promote HIF expression *in vitro *promote matrix accumulation and decrease degradation and hypertrophy, even in cells from OA joints.

## Introduction

The proper application of cellular therapies for articular cartilage repair is hindered by a lack of clarity with regard to the mechanisms that underlie the development and maintenance of the permanent chondrocyte phenotype as opposed to the transient endochondral phenotype. During endochondral ossification in development, transient chondrocytes of the anlagen undergo hypertrophy prior to apoptosis and replacement by bone. It is argued that hypertrophy is also a feature of osteoarthritis (OA) because the permanent chondrocytes of articular cartilage can express markers of hypertrophic endochondral chondrocytes, such as collagen X (*COL10A1*) and matrix metalloproteinase 13 (*MMP13*) during degeneration. The use of chondrocytes for cartilage repair requires *in vitro *expansion, a process that leads to dedifferentiation whereby the chondrocytes decrease expression of cartilage matrix genes such as aggrecan (*ACAN*) and collagen II (*COL2A1*), with collagen I (*COL1A1*) becoming the predominant collagen type [1]. Although it has long been established that these chondrocytes can be redifferentiated in three-dimensional culture [2], this may not be completely successful, because hypertrophy-related markers can also be upregulated, even in chondrocytes from nondiseased joints [3,4]. Therefore, achieving a better understanding of the factors regulating hypertrophy has important implications both for tissue engineering and for treatment of OA.

Although it is known that oxygen levels and the Per-Arnt-Sim (PAS) family of transcription factors known as hypoxia-inducible factors (HIFs), particularly HIF-1α and HIF-2α, play an active role in chondrocyte biology, their precise contributions to both cartilage maintenance and the progression of disease remain unclear [5-7]. These α-subunits are subject to degradation in the presence of sufficient oxygen, but in hypoxic environments such as the avascular joint, their stabilization allows heterodimerization and transactivation of hypoxia-responsive target genes. *In vitro *hypoxic culture of healthy human chondrocytes or cartilage explants causes an increase in HIF expression and promotes chondrogenic matrix genes [8-10] while suppressing MMP-1 and MMP-13 expression and activity [10,11] and decreasing *ADAMTS5 *mRNA expression and aggrecanase-mediated degeneration [10]. However, recent data suggest that HIF-2α can promote expression of genes involved in cartilage degeneration and hypertrophy [12,13]. The potential relevance of oxygen-dependent signaling to OA has been acknowledged for some time. In the past decade, multiple groups have reported elevated HIF-1α expression in degenerated cartilage [14-16]. However, two groups have independently identified numerous hypertrophic and OA-associated genes as targets of HIF-2α regulation, including *COL10A1 *and matrix metalloproteinases *MMP1*, *MMP3*, *MMP9 *and *MMP13*, using reporter gene assays [12,13]. These studies provoked considerable speculation about the importance of HIF-2α in hypertrophy and OA [17], but other studies have raised questions about its role in these processes [5,7]. For example, Araldi *et al. *found that conditional knockout of *Epas1 *(the HIF-2α gene) had minimal effects on *Col10a1 *expression [5]. As noted in a commentary on the initial HIF-2α/hypertrophy reports, the data are from *in vitro *overexpression studies conducted in 20% oxygen [6]. Because oxygen-dependent posttranslational regulation of HIFs is thought to be the primary means of control, such culture conditions may have influenced the observed downstream effects.

In our present study, we investigated the effects of oxygen on three-dimensional redifferentiation in both healthy and OA chondrocytes. The proteomes of dedifferentiated OA and healthy chondrocytes were reported to be differentially modulated by changes in oxygen tension using cells in monolayer culture [18] and more recently were shown to differentially respond to hypoxia when cultured with interleukin 1β (IL-1β) [19]; however, we are aware of no such comparison during three-dimensional redifferentiation published to date. Three-dimensional culture of chondrocytes in defined medium promotes matrix accumulation and redifferentiation [20]. Although this is considered preferable for chondrocytes that are to be implanted, it can also increase expression of hypertrophic genes. For example, Dehne *et al. *found that runt-related transcription factor 2 (*RUNX2*) and *COL10A1 *were both increased during redifferentiation, regardless of whether the cells were of nondiseased or osteoarthritic origin [3]. An increase in *COL10A1 *upon three-dimensional culture was also reported for chondrocytes from both total joint replacement knees and autologous chondrocyte implantation surplus cartilage [4]. Because three-dimensional culture can promote redifferentiation while also increasing markers of hypertrophy, this system allows a comparative analysis of the hypertrophic and degradative response that could be missed in monolayer culture. We hypothesized that this response might be differentially regulated by hypoxia in OA and healthy chondrocytes concomitant with different HIF expression patterns. To investigate this possibility, we evaluated healthy and OA chondrocytes' hypertrophic and degradative responses to hypoxia (2% oxygen), as well as the nature and quantity of matrix production and expression of HIFs.

## Materials and methods

### Isolation and expansion of human chondrocytes

Healthy chondrocytes were harvested from normal cadaver femoral condyles and OA chondrocytes from tissue taken during total joint replacement surgery (*n *= 5 each; for representative images, see Additional file 1). Human tissue was obtained as discard tissue without patient identifiers. The Oregon Health & Science University Institutional Review Board approved the study as being exempt from the requirement for consent. Cartilage was scraped from the condyles and finely minced before enzymatic digestion. Cartilage digestion was initiated with 1% protease (wt/vol) from *Streptomyces griseus *in low-glucose Dulbecco's modified Eagle's medium (DMEM; Life Technologies, Grand Island, NY, USA) supplemented with 1% penicillin-streptomycin (P/S). After 1 h at 37°C, protease was removed and replaced with 1,300 U/ml collagenase II (Worthington Biochemical, Lakewood, NJ, USA) in DMEM + P/S for 3 h at 37°C. The cell suspension was passed through a 40-μm cell strainer and centrifuged at 500 × *g *for 5 min, then the cells were resuspended in low-glucose DMEM supplemented with 10% fetal bovine serum and 1% P/S. Chondrocytes were plated at 7,000/cm^2 ^and expanded in monolayer culture in a standard tissue culture incubator with atmospheric oxygen and 5% CO_2_.

The storage conditions for osteochondral allografts may affect chondrocyte viability and metabolic activity, although the time at which these effects become evident depends on the storage solution, and in some medium solutions no change is observed even after 2 wk [21]. We obtained OA specimens just hours after total joint replacement, and healthy cartilage was collected from discarded osteochondral allografts of proprietary storage conditions. For the chondrocytes used here, however, we found no significant difference in either the number of viable cells obtained per gram of tissue digested (5.1 × 10^6 ^± 3.3 × 10^6 ^versus 6.2 × 10^6 ^± 3.1 × 10^6^, for healthy and OA cells, respectively) or in the doubling time from initial plating to first passage (6.9 ± 2.1 versus 6.4 ± 1.5 for healthy and OA cells, respectively).

### Pellet culture redifferentiation of chondrocytes

Chondrocytes were redifferentiated between the first and third passages with serum-free chondrogenic induction medium consisting of high-glucose DMEM (Life Technologies) containing 10 ng/ml transforming growth factor β1 (Peprotech, Rocky Hill, NJ, USA), 10^-7 ^M dexamethasone, 37.5 μg/ml ascorbic acid 2-phosphate, 1 mM sodium pyruvate, 40 μg/ml L-proline, 1 × ITS+ Universal Culture Supplement Premix (BD Biosciences, San Jose, CA, USA) and 1% P/S. Pellet cultures were formed by centrifuging 1 × 10^5 ^cells at 500 × *g *in 250 μl of medium in Nunc polypropylene V-bottom 96-well plates (Thermo Fisher Scientific, Waltham, MA, USA) and maintained in a hypoxic chamber (BioSpherix, Lacona, NY, USA) set at 2% oxygen, 5% CO_2 _or a standard tissue culture incubator with 5% CO_2_. Although CO_2 _displacement actually lowers the oxygen level, hereafter we refer to the standard condition as 20% oxygen according to convention. Medium was changed every 2 or 3 days, with that of the hypoxic cultures being done at 2% oxygen.

### Biochemical assays

All biochemical assays were conducted on at least triplicate pellets. Pellets were rinsed with phosphate-buffered saline (PBS) and digested overnight at 60°C in 4 U/ml papain (Sigma-Aldrich, St Louis, MO, USA) in PBS containing 6 mM Na_2_-ethylenediaminetetraacetic acid and 6 mM L-cysteine (papain buffer, pH 6.0). For samples to be assayed for hydroxyproline, iodoacetic acid was added to a final concentration of 10 mM after papain digestion.

DNA content of all papain-digested pellets was quantified using 2 μg/ml Hoechst dye with calf thymus DNA diluted in papain buffer used to prepare standard curves. Briefly, 50 μl of samples, standards and blanks were added to black 96-well plates with 200 μl of Hoechst dye before fluorescence emission was measured with a multiwell plate reader (excitation 355 nm, emission 455 nm).

The sulfated glycosaminoglycan (sGAG) content of pellets and culture medium was quantified using 1,9-dimethymethylene blue (DMMB) dye. Supernatant collected at each medium change was used to quantify the total amount of sGAGs produced and lost into the medium. Shark chondroitin sulfate diluted in either DMEM or papain buffer was used to prepare standard curves. For microplate assays, 50 μl of samples, standards and blanks were added to clear 96-well plates with 200 μl of DMMB dye (18 μg/ml in 0.5% ethanol, 0.2% formic acid, 30 mM sodium formate; pH 3.0) before absorbance was measured (575 nm).

Hydroxyproline content of pellets was quantified using an adaptation of the chloramine-T hydrate oxidation/p-dimethylaminobenzaldehyde development method with solid-phase hydrolysis on Dowex 50WX8-400 ion exchange resin (Thermo Fisher Scientific) [22]. *Trans*-4-hydroxy-L-proline in papain buffer was used to generate a standard curve. Absorbance of samples was measured at 560 nm in a multiwell plate reader.

### RNA isolation and real-time quantitative polymerase chain reaction

The RNeasy Mini Kit (QIAGEN, Germantown, MD, USA) was used to collect total RNA from four to six pellets of each condition. Pooled pellets were snap-frozen in liquid nitrogen and crushed, then immediately lysed with Buffer RLT lysis buffer (QIAGEN) containing 40 mM dithiothreitol (DTT). RNA isolation then proceeded as per the manufacturer's instructions.

RNA samples (250 ng) were reverse-transcribed using qScript cDNA SuperMix (Quanta BioSciences, Gaithersburg, MD, USA) as per the manufacturer's instructions. Quantitative polymerase chain reaction (qPCR) analysis was performed using a MyiQ iCycler thermal cycler (Bio-Rad Laboratories, Hercules, CA, USA) with PerfeCTa qPCR FastMix (Quanta BioSciences) and TaqMan assays (Life Technologies) for *ACAN *(Hs00153936_m1), *COL2A1 *(Hs00264051_m1), *COL1A1 *(Hs00164004_m1), *COL10A1 *(Hs00166657_m1), *MMP1 *(Hs00899658_m1), *MMP2 *(Hs00234422_m1), *MMP3 *(Hs00968305_m1), *MMP9 *(Hs00234579_m1), *MMP13 *(Hs00233992_m1), *MMP14 *(Hs00237119_m1), *TIMP2 *(Hs00234278_m1), *ADAMTS4 *(Hs00192708_m1), *ADAMTS5 *(Hs00199841_m1), *HAS2 *(Hs00193435_m1), *HIF1A *(Hs00153153_m1) and *EPAS1 *(Hs01026149_m1). The cycling parameters were 45°C for 2 min, 95°C for 1 min, then 95°C for 5 s and 60°C for 30 s for a total of 40 cycles. Results were analyzed using the 2^-ΔCt ^method relative to the housekeeping gene *18S *(Hs99999901_s1).

### Gelatin zymography

Gelatin zymography was performed on 10% SDS-PAGE gels containing 10 mg/ml gelatin. Gelatin was dissolved in 1.5 M Tris, pH 8.8, and displaced a corresponding volume of 1.5 M Tris during gel preparation. Medium samples from days 9 to 14 of pellet culture were pooled for each oxygen condition. Pooled samples were prepared in Lammeli sample buffer without reducing agents or boiling, and 20 μl of this solution was used for zymography. Samples were run alongside PageRuler Plus marker (Thermo Fisher Scientific), then gels were incubated in renaturing buffer (2.7% Triton X-100) two times for 30 min each before equilibrating in developing buffer (50 mM Tris base, 5 mM CaCl_2_, 40 mM HCl, 200 mM NaCl, 0.02% (wt/vol) Brij-35) for 30 min at room temperature. The developing buffer was then changed, and the gels were incubated at 37°C for 24 h. Developed zymograms were stained with Coomassie Blue (0.1% Coomassie R-250, 10% acetic acid, 40% methanol) for 45 min, then destained in a solution of 10% acetic acid and 20% methanol until bands were resolved.

### Immunohistochemical analysis

Pellets were fixed in 10% neutral buffered formalin, embedded in paraffin and sectioned onto slides. Sections were deparaffinized and treated with 1 mg/ml protease in PBS for 30 min at room temperature. For collagen I and collagen II double-staining, protease treatment was followed by 1 mg/ml hyaluronidase in PBS for 30 min at 37°C. Slides blocked with 5% bovine serum albumin (BSA) were probed overnight at 4°C with primary antibodies to collagen I (rabbit polyclonal antibody, 1:200; kind gift from A Hollander, University of Bristol, UK), collagen II (II-II6B3 mouse monoclonal antibody, 1:200; Developmental Studies Hybridoma Bank, University of Iowa, Iowa City, IA, USA), and collagen X (mouse polyclonal antibody, 1:300; kind gift from GJ Gibson, Henry Ford Hospital, Detroit, MI, USA) diluted in 1% BSA. Secondary antibodies (Oregon Green-conjugated anti-rabbit (1:250) and Alexa Fluor 596-conjugated anti-mouse (1:500) for double-staining; fluorescein isothiocyanate-conjugated anti-mouse diluted 1:500 for collagen X) were added in 1% BSA for 45 min at room temperature. Slides were mounted with ProLong Gold antifade reagent containing 4′,6-diamidino-2-phenylindole (Life Technologies) and imaged using an Olympus BX51 microscope (Olympus America, Melville, NY, USA).

### Western blot analysis

Whole-cell lysates were obtained by pooling 20 pellets from each condition at days 2, 7 and 14. Pellets were rinsed with PBS, snap-frozen in liquid nitrogen and crushed. The powdered sample was immediately resuspended in an enhanced radioimmunoprecipitation assay buffer with 1% SDS and 100 mM DTT [23]. Hypoxic cultures were maintained in low oxygen until directly before freezing.

Lysates were prepared in Laemmli sample buffer with 100 mM DTT and boiled for 3 min. Samples were run on 12% SDS-PAGE gels for histone H3 and on 6% SDS-PAGE gels for HIF blots using PageRuler Plus Prestained Protein Ladder (Molecular Weight Marker) (Thermo Fisher Scientific). Gels were transferred to polyvinylidene difluoride and blocked for 1 h with 2% milk-PBST (1 × PBS, 0.1% Tween 20) at room temperature. Primary antibody incubations were done in blocking solution overnight at 4°C. All washes were done three times for 10 min each in PBST.

The quantity of nuclear protein loaded between samples was normalized to histone H3. The relative signal of bands from a blot with equal volumes of lysates loaded were determined using an Odyssey imaging system (LI-COR Biosciences, Lincoln, NE, USA), and lysate volumes were adjusted accordingly for HIF protein blots. Anti-histone H3 primary antibody (ab24834; Abcam, Cambridge, MA, USA) was used at a 1:500 dilution, and IRDye 800CW secondary antibody (catalog no. 926-32210; LI-COR Biosciences) was used at a 1:400 dilution. HIF blots were probed with anti-HIF-1α (1:250 dilution, catalog no. 610958; BD Biosciences) and anti-HIF2α antibodies (1:500 dilution, sc-46691; Santa Cruz Biotechnology, Santa Cruz, CA, USA). To visualize blots, horseradish peroxidase-conjugated secondary antibody (1:2,500 dilution, catalog no. W4021; Promega, Madison, WI, USA) and Western Lightning Plus-ECL (PerkinElmer, Waltham, MA, USA) were used with a Kodak RP X-OMAT Developer (Eastman Kodak Health Imaging, Rochester, NY, USA).

### Statistical analysis

SPSS version 19.0 software (SPSS, Chicago, IL, USA) was used to assess statistical significance, which was defined as *P *< 0.05. Normality was evaluated using the Shapiro-Wilk test. Two-tailed independent *t*-tests or paired *t*-tests were used for normally distributed data, and Mann-Whitney *U *or Wilcoxon tests were used for nonparametric data. The unpaired tests were used to compare means for healthy chondrocytes with those of OA chondrocytes at a given oxygen level, and paired tests were used to compare means between oxygen levels within a given disease condition. The correlation coefficient, Pearson's *r*, for the ratios of mRNA expression of hyaluronic acid synthase 2 (*HAS2*) and sGAG retention at 2% oxygen over 20% oxygen was determined using bivariate analysis.

## Results

### Oxygen-dependent expression of cartilage matrix genes

**Figure 1:**
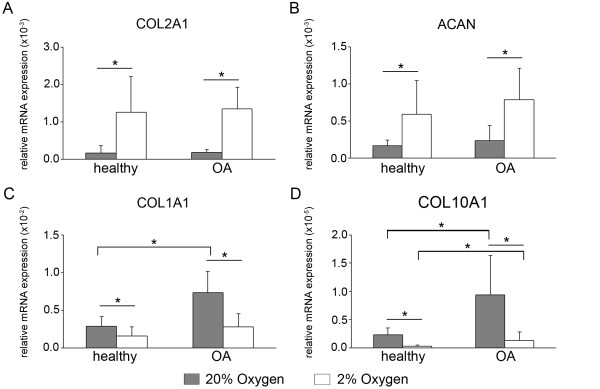
**Oxygen-dependent expression of cartilage matrix genes in healthy and osteoarthritic chondrocytes**. The relative gene expression levels of *COL2A1 ***(A)**, *ACAN ***(B)**, *COL1A1 ***(C) **and *COL10A1 ***(D) **in pellet cultures following 2 wk of redifferentiation in either 20% oxygen (gray bars) or 2% oxygen (white bars) were analyzed by real-time quantitative polymerase chain reaction. All values are the mean mRNA level normalized to 18S ribosomal RNA for chondrocyte pellets from *n *= 5 donors. Error bars represent 1 SD. Statistical significance was determined by performing independent *t*-tests, Mann-Whitney *U *test (between disease conditions) and paired *t*-test or Wilcoxon tests as appropriate (between oxygen levels). *P < 0.05.

When the expression levels of major matrix genes were compared, healthy and OA chondrocytes had similar responses: *COL2A1 *and *ACAN *(Figure 1A, B) were significantly higher at 2% compared with 20% oxygen, whereas *COL1A1 *and *COL10A1 *(Figures 1C and 1D) were significantly lower. Furthermore, the *COL2A1*: *COL1A1 *ratio was consistently increased by hypoxia compared to normoxia (9- to 87-fold in healthy pellets and 8- to 54-fold in OA pellets; Additional file 2). Because of the higher variability of the increase in healthy cell pellets, this was not significant at the α = 0.05 level (*P *= 0.097; *P *= 0.047 for OA). There was no significant difference in the *COL2A1*: *COL1A1 *ratio between healthy and OA cells at either oxygen level. No statistically significant differences were detected in expression levels between healthy and OA chondrocytes at day 0 of pellet cultures, and *COL10A1 *was undetectable (data not shown); however, redifferentiation led to differential changes in the two groups. *COL2A1 *and *ACAN *levels were similar between healthy and OA at both oxygen levels, but *COL1A1 *and *COL10A1 *were greater in OA chondrocyte pellets at 20% oxygen, a difference that was also significant for *COL10A1 *in 2% oxygen.

### Oxygen-dependent patterns of collagen distribution

**Figure 2:**
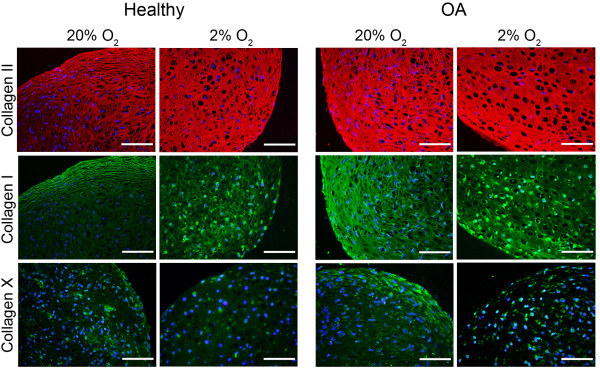
**Oxygen-dependent patterns of collagen distribution in healthy and osteoarthritic chondrocytes**. Representative images of matrix staining patterns are shown. Paraffin sections were either double-stained with anti-collagen I and anti-collagen II antibodies or single-stained with anti-collagen X as described in Materials and methods. 4′,6-diamidino-2-phenylindole was used as a counterstain for nuclei. Positive immunolocalization of collagen appears in red (collagen II) or in green (collagen I, collagen X), and nuclei are shown in blue. Negative controls with isotype-matched antibodies (images not shown) were used for background correction. Composite images of matrix staining and nuclei were digitally processed using ImageJ software (National Institutes of Health, Bethesda, MD, USA). Scale bars = 100 μm.

Regardless of the disease state, oxygen-dependent changes in matrix production and organization detected by immunohistochemical staining were similar (Figure 2). Although pellets from both oxygen levels stained positive for collagen II throughout the matrix, collagen I had distinct oxygen-dependent staining patterns. Pellets cultured at 20% oxygen had the most intense collagen I staining around the periphery of the pellets, where the cells also had a distinctly elongated (fusiform) morphology. All pellets cultured at 2% oxygen had qualitatively less collagen I staining in the matrix than 20% oxygen pellets, and they also lacked the flattened cells at their periphery. Additionally, the positive signal for collagen I in hypoxic pellets appeared to be intracellular. Collagen X was barely detectable in the matrices of both healthy and OA pellets, but, as with collagen I, the strongest staining was at the periphery of the pellets cultured at 20% oxygen.

### Oxygen-dependent matrix production

**Figure 3:**
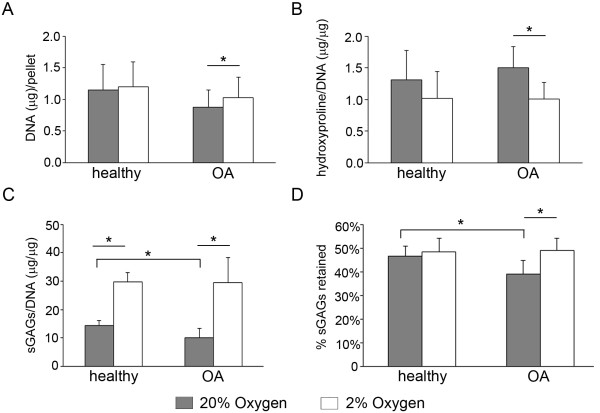
**Oxygen-dependent matrix production in healthy and osteoarthritic chondrocytes**. The DNA content per pellet **(A)**, hydroxyproline/DNA **(B)**, sulfated glycosaminoglycans (sGAGs)/DNA **(C) **and percentage of total sGAGs retained in the pellet matrix **(D) **were quantified for papain-digested pellets following 2 wk of redifferentiation in either 20% oxygen (gray bars) or 2% oxygen (white bars). All values are the means ± 1 SD for chondrocyte pellets from *n *= 5 donors. Statistical significance was determined by performing independent *t*-tests or Mann-Whitney U test (between disease conditions) and paired *t*-test or Wilcoxon tests as appropriate (between oxygen levels). *P < 0.05.

DNA content was used to normalize biochemical measures of matrix production. Although there was no difference between healthy and OA chondrocytes in DNA content at either oxygen level, OA pellets had significantly lower DNA when cultured in 20% oxygen than in 2% oxygen (*P *= 0.030) (Figure 3A). In healthy chondrocytes, DNA content was not significantly different between the two oxygen levels.

In both healthy and OA chondrocyte pellets, the mean hydroxyproline/DNA was higher in 20% oxygen compared with 2% oxygen (Figure 3B). In OA pellets, the difference in this measure between the two oxygen levels was significant. Despite the fact that, for each of the five healthy donors' chondrocytes, their 20% oxygen pellets contained higher hydroxyproline/DNA than their matched 2% oxygen cultures, this difference was not significant (*P *= 0.075). Hydroxyproline content was not significantly different between healthy and OA chondrocytes at either oxygen level.

Both healthy and OA chondrocytes had significantly more sGAG/DNA in pellets at 2% compared with those at 20% oxygen (Figure 3C). Although there was no significant difference between healthy and OA chondrocytes at 2% oxygen, OA pellets had significantly less sGAG/DNA than healthy counterparts at 20% oxygen (Figure 3C). Culture medium sGAG content was quantified, and the degree of proteoglycan retention was assessed, using sGAGs in pellets/total sGAGs produced (Figure 3D). Not only was the percentage of sGAGs retained within OA pellets significantly lower than that of healthy pellets in 20% oxygen, but the percentage retention of sGAGs was also found to be a significant measure of the oxygen response of healthy and OA chondrocytes. Although oxygen level did not affect retention of sGAGs in healthy chondrocyte pellets, OA pellets retained significantly less sGAGs at 20% oxygen.

### Oxygen-dependent expression of catabolic and anabolic enzymes

**Figure 4:**
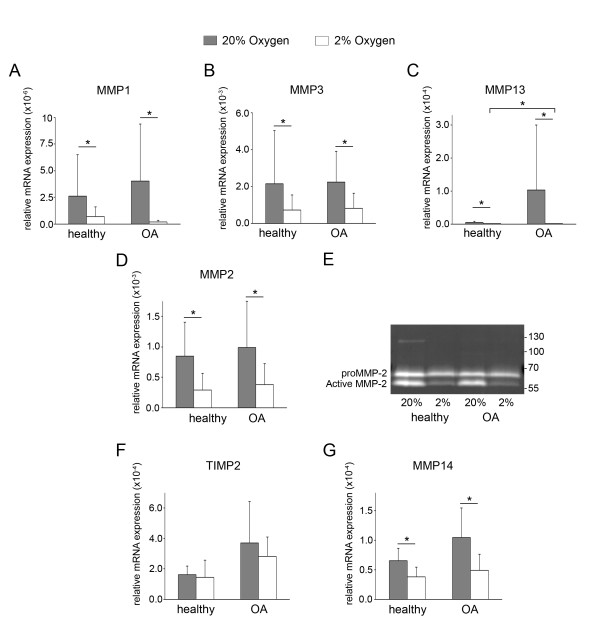
**Oxygen-dependent expression of matrix metalloproteinases in healthy and osteoarthritic chondrocytes**. **(A) **through **(D) **The relative gene expression levels of *MMP1*, *MMP3*, *MMP13 *and *MMP2 *in pellet cultures following 2 wk of redifferentiation in either 20% oxygen (gray bars) or 2% oxygen (white bars) were analyzed by real-time quantitative polymerase chain reaction. **(E) **Gelatin zymography of supernatants pooled from days 9 to 14 of pellet cultures indicates levels of secreted pro-MMP2 and active MMP2 in healthy and osteoarthritic (OA) cells at 20% and 2% oxygen. **(F) **and **(G) **The relative gene expression levels of *TIMP2 *and *MMP14 *in pellet cultures following 2 wk of redifferentiation in either 20% oxygen (gray bars) or 2% oxygen (white bars) were analyzed by real-time quantitative polymerase chain reaction. All expression values are the mean mRNA levels normalized to 18S ribosomal RNA for chondrocyte pellets from *n *= 5 donors. Error bars represent 1 SD. Statistical significance was determined by performing independent *t*-tests (between disease conditions) and paired *t*-tests (between oxygen levels). *P < 0.05.

Expression of *MMP1*, *MMP3 *and *MMP13 *(Figures 4A through 4C) were all significantly lower in 2% oxygen than in 20% oxygen cultures for both healthy and OA chondrocytes. *MMP13 *gene expression was both oxygen- and disease state-dependent. At 20% oxygen, very high *MMP13 *gene expression levels seen in some OA samples resulted in a mean 22-fold difference between OA and healthy chondrocytes, but the wide variation meant that statistical significance was not established (*P *= 0.123). At 2% oxygen, however, *MMP13 *was significantly higher in OA than in healthy chondrocytes (19-fold). Although these differences were observed in redifferentiated pellets, there was no statistically significant difference in *MMP13 *expression between healthy and OA cells at day 0 of pellet culture (data not shown).

Like the other MMPs, *MMP2 *was significantly lower in 2% oxygen than in 20% oxygen for both healthy and OA chondrocytes (Figure 4D). Furthermore, gelatin zymography indicated that less of the active form of MMP2 was generated at 2% oxygen (Figure 4E). Additionally, supernatants from healthy cells in 20% oxygen displayed unidentified gelatinolytic activity near 130 kDa. We could not consistently detect *MMP9 *mRNA expression at either oxygen level in either healthy or OA cells (data not shown), and supernatants did not show gelatinolytic activity in the molecular weight range where MMP9 is typically detected (92 kDa).

Since MMP2 displayed oxygen-dependent generation of its active form, we evaluated expression of the two essential members of the complex responsible for activation of pro-MMP2 at the cell surface, tissue inhibitor of *MMP 2 *(*TIMP2*) and *MMP14 *(Figures 4F and 4G). Although *TIMP2 *was not oxygen-dependent, like the other MMPs evaluated, *MMP14 *was expressed at a significantly lower level in 2% oxygen in both healthy and OA cells.

**Figure 5:**
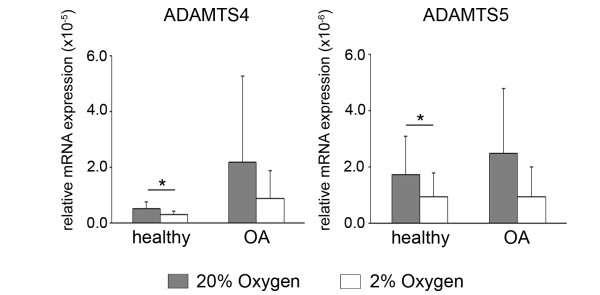
**Oxygen-dependent expression of aggrecanases in healthy and osteoarthritic chondrocytes**. The relative gene expression levels of *ADAMTS4 ***(A) **and *ADAMTS5 ***(B) **in pellet cultures following 2 wk of redifferentiation in either 20% oxygen (gray bars) or 2% oxygen (white bars) were analyzed by real-time quantitative polymerase chain reaction. All values are the mean mRNA levels normalized to 18S ribosomal RNA for chondrocyte pellets from *n *= 5 donors. Error bars represent 1 SD. Statistical significance was determined by performing independent *t*-tests (between disease conditions) and paired *t*-tests (between oxygen levels). *P < 0.05.

Neither aggrecanase 1 (*ADAMTS4*) nor aggrecanase 2 (*ADAMTS5*) (Figures 5A and 5B) had disease state-dependent expression, regardless of the oxygen level. However, expression of both genes showed some degree of oxygen-dependent regulation. Both *ADAMTS4 *and *ADAMTS5 *were significantly higher in healthy chondrocytes at 20% compared with 2% oxygen. Whereas their expression was higher at 20% oxygen for both genes in four of the five OA samples, this difference was not significant (*P *= 0.119 and *P *= 0.127, respectively).

**Figure 6:**
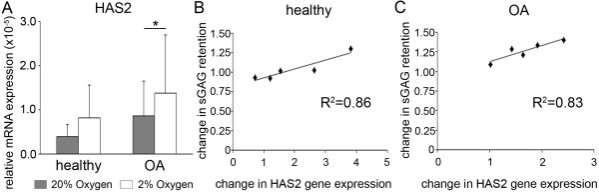
**Oxygen-dependent expression of hyaluronic acid synthase 2 (*HAS2*) in healthy and osteoarthritic chondrocytes and correlation with sulfated glycosaminoglycan retention**. **(A) **The relative gene expression levels of *HAS2 *in pellet cultures following 2 wk of redifferentiation in either 20% oxygen (gray bars) or 2% oxygen (white bars) was analyzed by real-time quantitative polymerase chain reaction. All values are the mean mRNA levels normalized to 18S ribosomal RNA for chondrocyte pellets from *n *= 5 donors. Error bars represent 1 SD. Statistical significance was determined by performing independent *t*-tests (between disease conditions) and paired *t*-tests (between oxygen levels). *P < 0.05. **(B) **and **(C) **The oxygen-dependent change in sulfated glycosaminoglycan (sGAG) matrix retention (percentage retained at 2% oxygen/percentage retained at 20% oxygen) was plotted against the oxygen-dependent changes in *HAS2 *expression (*HAS2 *at 2% oxygen/*HAS2 *at 20% oxygen) for healthy (B) and osteoarthritic (OA) pellets (C). Linear regression fit and coefficient of determination (*R*^2^) are shown. Bivariate analysis revealed a strong and statistically significant correlation between the oxygen-dependent difference in *HAS2 *and that of sGAG retention in both OA cells (*P *= 0.032) and healthy cells (*P *= 0.023).

In addition to genes involved in modulating degradation, we evaluated expression of *HAS2 *(Figure 6A), a candidate enzyme for improving proteoglycan retention through hyaluronic acid production. *HAS2 *was significantly higher in hypoxic conditions in OA chondrocytes; in healthy cells, though it trended in the same direction, the difference in expression between 2% and 20% oxygen was nonsignificant (*P *= 0.150). As with *ADAMTS4 *and *ADAMTS5 *in the OA cells, *HAS2 *was lower in 20% oxygen in all but one of the healthy donors' chondrocyte pellets. Additionally, bivariate analysis revealed a strong and statistically significant correlation between the oxygen-dependent difference in *HAS2 *and that of sGAG retention in both OA cells (Pearson's *r *= 0.909, *P *= 0.032) (Figure 6B) and healthy cells (Pearson's *r *= 0.927, *P *= 0.023) (Figure 6C). No significant correlation was found for either *ADAMTS4 *or *ADAMTS5*.

### HIF-1α and HIF-2α expression during chondrocyte redifferentiation

**Figure 7:**
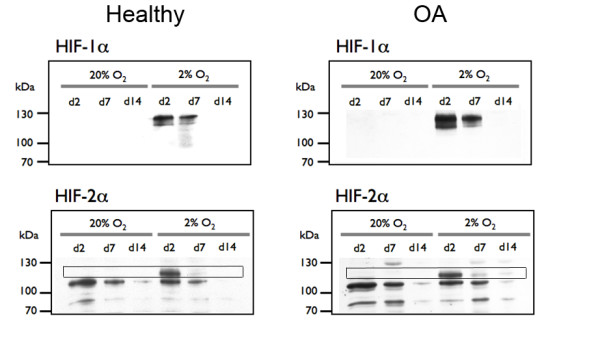
**Hypoxia-inducible factors 1α and 2α protein expression during redifferentiation of healthy and osteoarthritic chondrocytes**. Representative images of banding patterns are shown. Normalization of nuclear protein loading was achieved by first running equal volumes of each sample and probing for histone H3. For hypoxia-inducible factor (HIF) blots, the volume loaded was adjusted to give equivalent nuclear loading based on each histone H3 band's signal relative to the signal from the lowest-intensity band. The location of the only oxygen-dependent band on HIF-2α blots is in the boxed area, and the blot is cropped to show only the bands in the molecular weight range where the HIF-2α product is predicted (115 kDa). Unmodified HIF-2α blots for healthy and OA chondrocytes are shown in Additional files 3 and 4, respectively. The consistency of the nonspecific band near 35 kDa (lowest band) was used as a loading control after histone H3 normalization.

Representative images of the patterns of HIF-1α and HIF-2α expression for chondrocyte pellets are shown in Figure 7. Expression patterns of both HIF-1α and HIF-2α in healthy and OA chondrocytes were indistinguishable. This was consistent with mRNA data for *HIF1 *and *EPAS1*, which showed no significant differences between healthy and OA cells at day 0 or at day 14, regardless of oxygen level (data not shown). Little to no HIF-1α or HIF-2α was detected throughout 20% oxygen cultures. In contrast, both healthy and OA chondrocytes expressed HIF-1α and HIF-2α at 2% oxygen, with the strongest signals occurring early (day 2). When day 2 protein from hypoxic pellets was run on the same gel, we could not determine a disease-dependent difference in band intensity for either HIF-1α or HIF-2α (data not shown). In addition to the bands detected at the predicted molecular weights, there were other positive bands on the immunoblots. For HIF-1α, the additional bands were determined to be specific, with a λ-phosphatase digestion indicating that they were due to phosphorylation (data not shown). For HIF-2α, only the band of the predicted molecular weight had oxygen-dependent expression; the identities of the other bands could not be confirmed (Figure 7, Additional file 3 and Additional file 4).

## Discussion

In this study, we evaluated the oxygen-dependent response of healthy and OA chondrocytes during redifferentiation. We hypothesized that OA chondrocytes might have a different response to changes in oxygen levels compared with healthy chondrocytes, such that reduced oxygen would promote hypertrophic and degenerative changes in OA chondrocytes concomitant with different HIF expression patterns. However, our data indicate that redifferentiation was promoted, hypertrophic and OA-associated degradative genes were suppressed and HIF expression patterns were indistinguishable in both healthy and OA chondrocytes in hypoxic conditions.

Healthy and OA chondrocytes both responded with higher *COL2A1 *and *ACAN *and lower *COL1A1 *expression in hypoxic conditions. Hypoxic culture has previously been found to increase *COL2A1 *and *ACAN *in nondiseased chondrocytes [8,9] and independently in chondrocytes from joint arthroplasties [24]. In a direct comparison, we found no differences in expression of these genes between healthy and diseased cells, regardless of oxygen tension. Additionally, though *COL1A1 *was higher in OA chondrocytes than in healthy chondrocytes at 20% oxygen, lower *COL1A1 *and less collagen I matrix staining was associated with all cells redifferentiated in hypoxia. Collagen prolyl hydroxylases require molecular oxygen, and the decreases in hydroxyproline content at 2% oxygen indicate that this level of hypoxia impairs the activity of these enzymes. It is possible that, when oxygen levels are low enough that these enzymes are impaired in chondrocytes, prolyl hydroxylation and thus secretion of collagen type II is preferentially maintained over other collagens. Consistent with this theory, the *COL2A1*: *COL1A1 *ratio was consistently elevated by hypoxia in our studies.

We also found that hypoxia increased proteoglycan accumulation by both healthy and OA cells. Excess oxygen may be more detrimental to cells from OA joints, however, because OA cell pellets had significantly less sGAG/DNA than those of healthy cells maintained at high oxygen. Whether this is due to a greater oxygen sensitivity that results in impaired synthesis, more degradation, disturbed matrix assembly or some combination of these processes remains to be determined. The percentage of sGAGs retained within the matrix was also significantly reduced in OA chondrocytes at 20% oxygen. This may be indicative of poorer proteoglycan incorporation into the matrix or of increased degradation. With regard to the former possibility, we assessed expression of *HAS2*, the predominant form of the HAS enzyme in chondrocytes [25,26]. Previously, studies using RNA interference against *HAS2 *showed that this did not affect *ACAN *expression or proteoglycan synthesis, but that it did decrease the amount of proteoglycans retained within the matrix [25]. *HAS2 *expression and hyaluronic acid synthesis have also been shown to be oxygen-dependent in bovine chondrocytes [27]. We found that 20% oxygen resulted in lower mean *HAS2 *expression in both healthy and OA chondrocytes. Furthermore, there was a strong correlation between the oxygen-dependent difference in *HAS2 *and that of sGAG retention in both healthy and OA cells. Reduced hyaluronic acid synthesis could allow for more passive loss of proteoglycans; however, hyaluronic acid has also been reported to modulate expression and/or activity of ADAMTS4 [28] and MMP13 [29] in chondrocytes. Determining specific mechanisms by which *HAS2 *modulation by oxygen affects chondrocytes and their matrix requires further investigation.

The potent anabolic effects of hypoxia on chondrocytes are well-documented, but less is known about its regulation of catabolism. In the two studies implicating HIF-2α in hypertrophy and OA, *MMP3*, *MMP9/Mmp9 *and *MMP13 *were reported to be upregulated by HIF-2α [12,13]. One of the studies also reported *MMP1 *and *Adamts4*, but not *Adamts5*, to be promoted by HIF-2α [13], but the other found no association for either *Adamts4 *or *Adamts5*. In contrast, hypoxia reportedly decreases *MMP1 *and *MMP13 *expression and collagenolytic activity in healthy human chondrocytes and decreases *ADAMTS5 *and aggrecanase activity in healthy human cartilage explants [10,11]. We found that *MMP1 *and *MMP13 *were lower in hypoxia not only in healthy chondrocytes but also in OA chondrocytes. Additionally, *MMP2 *and *MMP3 *were lower in both healthy and OA chondrocytes in hypoxic culture, and less of the active form of MMP-2 was generated, concomitant with lower expression of *MMP14*. As reported by Ströbel *et al*., we detected no oxygen-dependent changes in *MMP9 *expression, because this gene was at the limit for detection in both healthy and OA cells [11]. *ADAMTS4 *and *ADAMTS5 *were also lower in both healthy and OA chondrocytes in hypoxia, but the differences were significant only in healthy cells. Although hypoxia was associated with transcriptional downregulation of these enzymes, we did not address the potential for oxygen-dependent activity. Like MMPs, the ADAMTS proteases are synthesized as inactive zymogens and assessing whether and to what extent their activation is oxygen-dependent is an essential next step in understanding the role of oxygen in cartilage degeneration. Recent findings by Thoms *et al. *indicate that hypoxic culture does indeed suppress loss of aggrecanase-generated aggrecan fragments from healthy cartilage explants and that *TIMP3 *may play a role in the oxygen-dependent inhibition [10].

We found that *COL10A1 *was dramatically lower at 2% oxygen compared with 20% oxygen in both healthy and OA chondrocytes. This finding contrasts with that of Schrobback *et al*., who reported that *COL10A1 *was not oxygen-dependent in chondrocytes from patients undergoing arthroplasty [30]. However, they used 5% oxygen, and, though it was reported as nonsignificant, *COL10A1 *was on average lower at 5% oxygen than at 20% oxygen. Because our data suggest that OA chondrocytes may be particularly sensitive to oxygen, the different oxygen levels could explain the discrepancies. Compared to standard culture conditions, human neuroblastoma cells grown at 5% oxygen were reported to have elevated levels of HIF-2α only, whereas those grown at 1% oxygen had increased levels of both HIF-1α and HIF-2α [31]. Although the relative contributions of the HIFs to collagen type expression remain unknown, we observed increased levels of both HIF-1α and HIF-2α at 2% oxygen. If expression patterns of the HIFs vary in chondrocytes at higher oxygen levels, this could alter collagen expression profiles.

Our finding that hypoxia decreases *COL10A1 *also seems to be at odds with results reported by Saito *et al. *indicating that HIF-2α was a potent transactivator of *COL10A1*, at least when paired with the β-subunit of ARNTL [12]. This discrepancy is not inexplicable, because it is known that HIFs are posttranslationally modified in a number of ways that may be dependent on culture conditions, including oxygen tension, and we performed our studies in hypoxic conditions, whereas Saito *et al. *did theirs at 20% oxygen. For example, both HIF-1α [32,33] and HIF-2α [34] have been reported to be phosphorylated in hypoxia. Although we cannot confirm the presence of HIF-2α modifications, phosphorylation of HIF-1α appears to be relevant in chondrocytes. The overexpression studies conducted in standard oxygen conditions could alter such regulatory mechanisms of HIF signaling and contribute to reported differences. With the reports of HIF-2α's involvement in promoting hypertrophic genes came suggestions that targeting HIF-2α could be an effective treatment for OA [17]. As was also noted, targeting the relevant dimerization partners involved in promoting these genes may be a more suitable strategy. Our results support this alternate view, because the absence of HIF expression in human chondrocytes is associated with increased markers of hypertrophy and degeneration and the banding patterns of the HIF α-subunits were identical, regardless of the cells' origin.

Although there were no obvious differences in the response of HIF-1α and HIF-2α expression between healthy and OA cells, because more of the measured outcomes were affected by high oxygen in OA chondrocytes, we cannot rule out that there are differences in oxygen-dependent signaling in OA chondrocytes compared with healthy chondrocytes. With higher oxygen exposure, OA chondrocytes have significantly higher *COL1A1 *and *COL10A1 *mRNA expression compared with healthy chondrocytes and exhibit loss of proteoglycans from the matrix in addition to having lower cell numbers at the end of the culture period compared with their hypoxic counterparts. Although there were differences between redifferentiated healthy and OA chondrocytes that were evident or more pronounced only under higher oxygen tension, only the expression levels of *COL10A1 *and *MMP13 *were significantly different between healthy and OA cells under hypoxic conditions. There may be HIF signaling-related mechanisms that are responsible for the disease state-dependent differences we see in certain parameters of redifferentiation. The α-subunits are generally assumed to be the rate-limiting factors because they are specific to hypoxic targets, unlike the promiscuous β-subunits. However, we cannot rule out that the presence and relative ratios of the β-subunits and control of dimerization could affect HIF activity in ways that would not be detected by Western blot analysis for HIF α-subunits. In addition, the asparginyl hydroxylase factor inhibiting HIF, which blocks coactivator recruitment, may be differentially regulated. Furthermore, epigenetic phenomena have been shown to be involved in the regulation of HIF target gene expression in chondrocytes. Hashimoto *et al. *demonstrated that methylation of CpG sites in the hypoxia-responsive element in the proximal promoter region of *MMP13 *inhibited HIF-2α-dependent transactivation [35]. This is an intriguing line of inquiry and may provide a valuable key to understanding the patterns of gene expression we describe and possibly even a mechanism for the donor-to-donor variation observed with primary cells.

## Conclusions

Hypoxic culture lessens the differences between OA and healthy chondrocytes, and the effect of elevated oxygen leads to changes in both healthy and OA chondrocytes that are generally associated with degenerative cartilage. This implies that a loss of HIF signaling could be involved in promoting changes associated with cartilage degradation. However, these studies were done in the absence of external stress signals, such as inflammatory factors, which could modulate HIF signaling and alter the downstream effects. That the detrimental effects of high oxygen are greater in the OA chondrocytes indicates that diseased cells may be less equipped to mitigate the stress of impaired HIF signaling and that this difference is maintained through *in vitro *expansion.

## Abbreviations

*ACAN*: human aggrecan gene; *Adamts4*/*ADAMTS4*: mouse/human aggrecanase 1 gene; *Adamts5*/*ADAMTS5*: mouse/human aggrecanase 2 gene; BSA: bovine serum albumin; *COL1A1*: human collagen type I, α1 gene; *COL2A1*: human collagen type II, α1 gene; *COL10A1*: human collagen type X, α1 gene; DMEM: Dulbecco's modified Eagle's medium; DMMB: 1,9-dimethymethylene blue; *Epas1*/*EPAS1*: mouse/human endothelial Per-Arnt-Sim domain-containing protein 1 (HIF-2α) gene; *HAS2*: human hyaluronic acid synthase 2 gene; HIF: hypoxia-inducible factor; MMP: matrix metalloproteinase; OA: osteoarthritis; PAS: Per-Arnt-Sim domain; P/S: penicillin-streptomycin; sGAG: sulfated glycosaminoglycan; *TIMP2*: human tissue inhibitor of metalloproteinases 2 gene.

## Competing interests

The authors declare that they have no competing interests.

## Authors' contributions

BDM participated in study conception and design and data interpretation, coordinated the experiments, acquired data (biochemistry, qPCR and immunohistochemistry), performed the statistical analyses and drafted the manuscript. HC participated in study conception and design, data acquisition and interpretation (immunohistochemistry, zymography and Western blotting) and helped to draft and critically revise the manuscript. BJ participated in study conception and design and data interpretation and critically revised the manuscript. All authors read and approved the final manuscript.

## Supplementary Material

Additional file 1 **Macroscopic and toluidine blue-stained sections of healthy and osteoarthritic cartilage specimens**. Representative cartilage specimen images of healthy **(A) **and **(C**) and OA **(B) and (D) **are shown. Click here for file

Additional file 2 **Ratio of *COL2A1 *to *COL1A1 *expression in healthy and osteoarthritic chondrocytes**. The ratios of the relative gene expression levels of *COL2A1 *and *COL1A1 *in pellet cultures following 2 wk of redifferentiation in either 20% oxygen (gray bars) or 2% oxygen (white bars) were calculated. All values are the mean ratios from *n *= 5 donors. Error bars represent 1 SD. Statistical significance was determined by performing independent *t*-tests (between disease conditions) and paired *t*-tests (between oxygen levels). *P < 0.05. Click here for file

Additional file 3 **Full hypoxia-inducible factor 2α blot for healthy chondrocytes shown in Figure 7**. Unmodified version of healthy chondrocyte HIF-2α blot shown in Figure 7. Click here for file

Additional file 4 **Full hypoxia-inducible factor 2α blot for osteoarthritic chondrocytes shown in Figure 7**. Unmodified version of OA chondrocyte HIF-2α blot shown in Figure 7. Click here for file
