# Supplementary figures and images for: Hypoxia promotes redifferentiation and suppresses markers of hypertrophy and degeneration in both healthy and osteoarthritic chondrocytes
Source: Arthritis Res Ther. 2013 Aug 21;15(4):R92. doi: 10.1186/ar4272 (PMC3979022; doi:10.1186/ar4272)

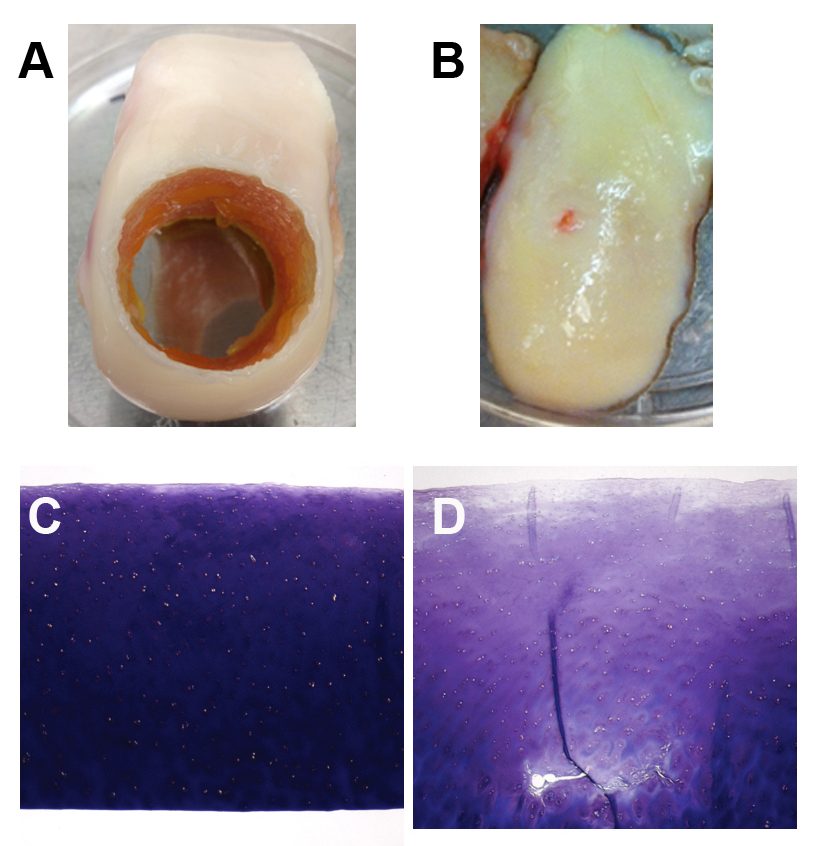

Supplement: Additional file 1 — Macroscopic and toluidine blue-stained sections of healthy and osteoarthritic cartilage specimens. Representative cartilage specimen images of healthy (A) and (C) and OA (B) and (D) are shown. [file ar4272-S1.JPEG]

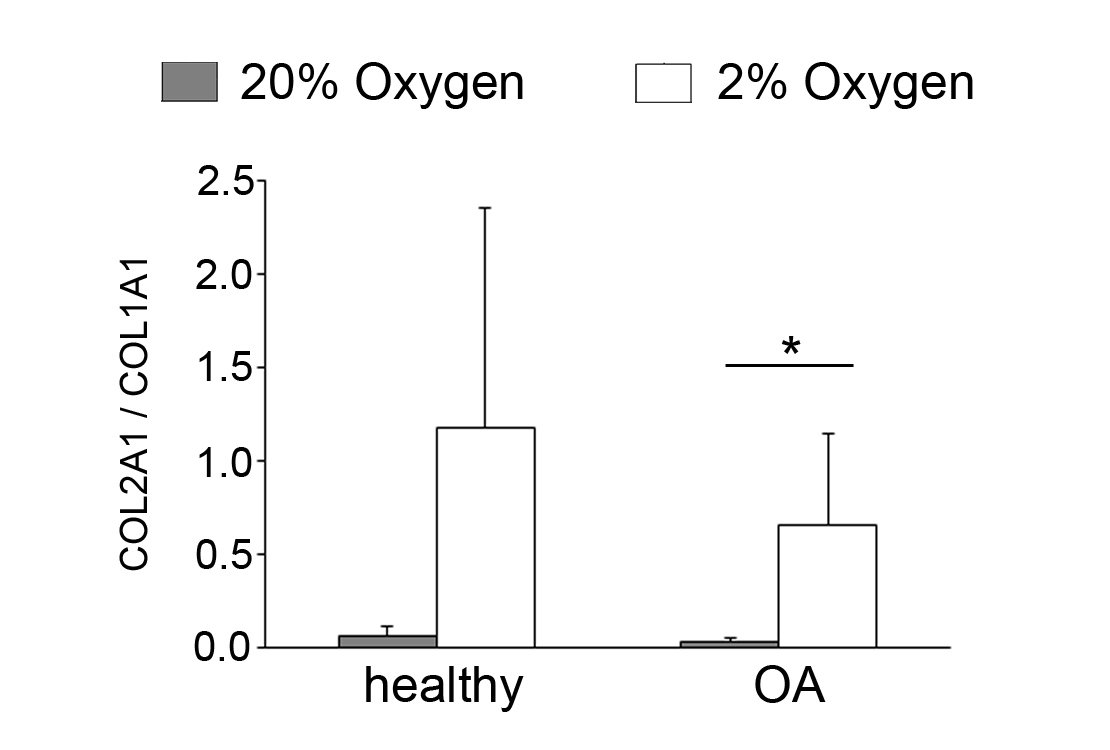

Supplement: Additional file 2 — Ratio of COL2A1 to COL1A1 expression in healthy and osteoarthritic chondrocytes. The ratios of the relative gene expression levels of COL2A1 and COL1A1 in pellet cultures following 2 wk of redifferentiation in either 20% oxygen (gray bars) or 2% oxygen (white bars) were calculated. All values are the mean ratios from n = 5 donors. Error bars represent 1 SD. Statistical significance was determined by performing independent t-tests (between disease conditions) and paired t-tests (between oxygen levels). *P < 0.05. [file ar4272-S2.TIFF]

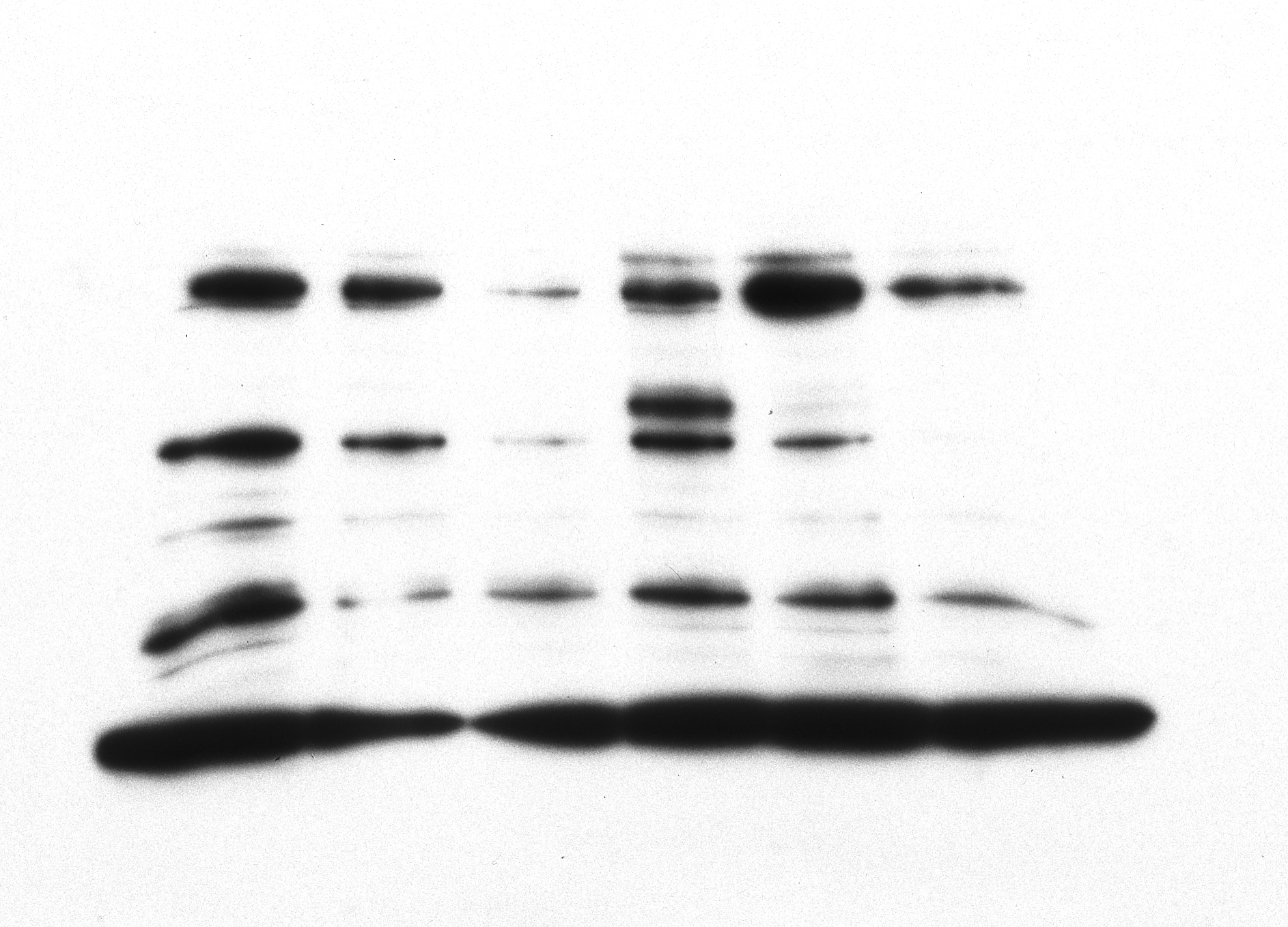

Supplement: Additional file 3 — Full hypoxia-inducible factor 2α blot for healthy chondrocytes shown in Figure 7. Unmodified version of healthy chondrocyte HIF-2α blot shown in Figure 7. [file ar4272-S3.TIFF]

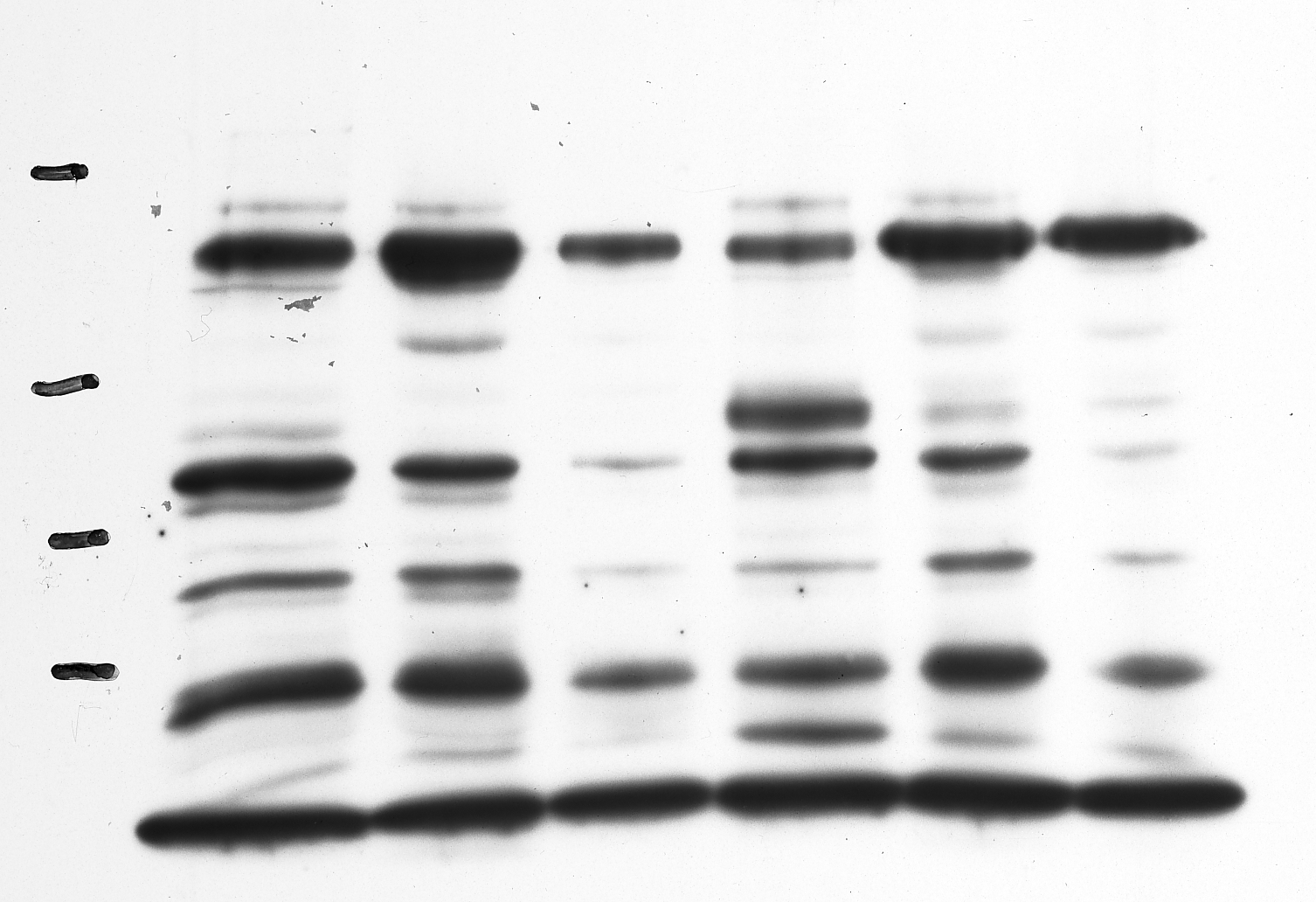

Supplement: Additional file 4 — Full hypoxia-inducible factor 2α blot for osteoarthritic chondrocytes shown in Figure 7. Unmodified version of OA chondrocyte HIF-2α blot shown in Figure 7. [file ar4272-S4.TIFF]
